# Supplementary material for: Notch1 promotes ordered revascularization through Semaphorin 3g modulation of downstream vascular patterning signalling factors
Source: J Physiol. 2022 Jan 17;600(3):509–30. doi: 10.1113/JP282286 (PMC9305962; doi:10.1113/JP282286)
Supplement: Supplementary file 2 — Statistical Summary Document [file TJP-600-509-s002.docx]

**Manuscript Title:** Notch1 Promotes Revascularization through Modulation of Downstream Vascular Patterning Signaling

**Authors:** James Hyun, Monica Lee, Jalees Rehman, Kostandin V. Pajcini, Asrar B. Malik

**Animal model used, if applicable:** Mouse –Transgenic (DNMAML^f/f^-Cdh5CRE^ERT2^) on C57Bl/6J background

Mouse –Transgenic (NICD^f/f^-Cdh5CRE^ERT2^) on C57Bl/6J background

Mouse –Transgenic (TdTomato^f/f^-Cdh5CRE^ERT2^) on C57Bl/6J background

**Underlying hypothesis:** This investigation tests the hypothesis that the modulation of Notch signaling effects collateral artery expansion through a Sema3g dependent mechanism. Notch signaling was inhibited through the overexpression of DNMAML and enhanced using NICD in endothelial specific Cre drivers before occlusion of the femoral artery in a mouse model of hindlimb ischemia.

**Definitions of ‘n’:**

Question 1: n = individual mouse tracked over the course of 14 days following hind limb ischemia

Question 2: n = individual collateral arteries from 5 separate animals of the specified genotype.

Question 3: n = individual animal from which endothelial cells were sorted from

Question 4: n = individual mouse tracked over the course of 14 days following hind limb ischemia from 8 separate animals of the specified genotype

Question 5: n = individual collateral arteries from 5 separate animals of the specified genotype from which multiple tissue sections were stained and observed.

Question 6: n = individual animal from which endothelial cells were sorted from the specified genotype

Question 7: n = individual animal from which endothelial cells were sorted from the specified genotype

Question 8: n = individual mouse tracked over the course of 14 days following hind limb ischemia from 8 separate animals of the specified genotype

Question 9: n = individual collateral arteries from 5 separate animals of the specified genotype from which multiple tissue sections were stained and observed.

Question 10: n = individual animal from which endothelial cells were sorted from the specified genotype

Question 11: n = individual animal from which endothelial cells were sorted from the specified genotype

Question 12: n = individual animal from which endothelial cells were sorted from the specified genotype

Question 13: n = number of separate experiments performed

Question 14: n = number of random spheroids imaged for analysis

Question 15: n = number of random spheroids imaged for analysis

Question 16: n = number of random spheroids imaged for analysis

Question 17: n = number of separate experiments performed

Question 18: n = number of separate experiments performed

Question 19: n = number of fields of view, 4 fields of view were imaged for analysis and were from 3 retinas of 3 different mice

Question 20: n = number of fields of view, 4 fields of view were imaged for analysis and were from 3 retinas of 3 different mice

Question 21: n = number of fields of view, 4 fields of view were imaged for analysis and were from 3 retinas of 3 different mice

Question 22: n = number of fields of view, 4 fields of view were imaged for analysis and were from 3 retinas of 3 different mice

**Statistical summary table:**

| Experimental question number* | Finding/ conclusion | Experimental location/ variable  e.g. muscle, neocortex or genotype | Mean value | SD | n val. | P** | Units | Data comparisons  e.g. WT vs KO | Statistical test | Any other variable  e.g. subjects’ age or sex | Figure/ table in which data are presented | Comments  e.g. observation |
| --- | --- | --- | --- | --- | --- | --- | --- | --- | --- | --- | --- | --- |
| 1. Assess perfusion recovery | Perfusion increases within 7days following HLI | Comparison of WT hindlimb | - | - | 5 | - | PU  Perfusion units | - | Observation | Days 1, 3, 5, 7, 14 | 1C | observation |
|  |  |  | 5.596 | 0.6980 | 5 | - | PU | - | Observation | Day1 | 1C |  |
|  |  |  | 9.978 | 2.4062 | 5 | - | PU | - | Observation | Day3 | 1C |  |
|  |  |  | 25.584 | 4.6108 | 5 | - | PU | - | Observation | Day5 | 1C |  |
|  |  |  | 35.872 | 6.0251 | 5 | - | PU | - | Observation | Day7 | 1C |  |
|  |  |  | 43.934 | 6.1089 | 5 | - | PU | - | Observation | Day14 | 1C |  |
| 2. Measure collateral artery diameter following HLI | Collateral arteries expand following HLI | WT non-ischemic adductor | 30.4083 | 8.5101 | 5 | **-** | μm | WT non-ischemic to ischemic | Students’ t test, unpaired | Day7 | 1E | IHC expts. Used validated Abs. Representative images were used in manuscript |
|  |  | WT ischemic adductor | 63.7777 | 14.0507 | 5 | **0.0031** | μm |  |  |  |  |  |
| 3. qRT-PCR of Notch targets relative to 18s post HLI | Notch1 and Hes1 increases following HLI | Compare ischemic to non-ischemic ECs  *Notch1* | 2.8101 | 0.1236 | 3 | 0.004 | Fold change | WT ischemic to non-ischemic | Students’ t test, unpaired | Day5 sorted EC | 1F |  |
|  |  | *Dll4* | 1.1111 | 0.2378 | 3 | - |  |  |  |  |  |  |
|  |  | *Jag1* | 3.9072 | 1.8514 | 3 | - |  |  |  |  |  |  |
|  |  | *Hes1* | 2.0819 | 0.0951 | 3 | **0.0151** |  |  |  |  |  |  |
|  |  | *Hey1* | 0.4125 | 0.4138 | 3 | - |  |  |  |  |  |  |
| 4. Assess perfusion recovery in DNMAML mice | DNMAML  reduces perfusion post HLI | Comparison of perfusion in WT vs DNMAML hind limbs | - | - | - | - | PU  Perfusion units | WT vs DNMAML | Multiple unpaired t-test. Holm-Sidak method | Days 1, 3, 5, 7 | 2C | observation |
|  |  | WT | 7.7434 | 1.3943 | 8 | 0.0692 | PU | + | + | Day1 | 2C |  |
|  |  | DNMAML | 9.5210 | 3.4286 | 8 |  | PU |  |  |  |  |  |
|  |  | WT | 15.3787 | 4.1514 | 8 | 0.0436 | PU | + | + | Day3 | 2C |  |
|  |  | DNMAML | 11.1279 | 4.7492 | 8 |  | PU |  |  |  |  |  |
|  |  | WT | 27.8435 | 9.7792 | 8 | 0.1249 | PU | + | + | Day5 | 2C |  |
|  |  | DNMAML | 19.6965 | 7.4624 | 8 |  | PU |  |  |  |  |  |
|  |  | WT | 44.1275 | 8.5071 | 8 | **0.000014** |  | + | + | Day7 | 2C |  |
|  |  | DNMAML | 24.4661 | 4.1812 | 8 |  |  |  |  |  |  |  |
| 5. Measure DNMAML collateral artery diameter following HLI | DNMAML  arteries failed to expand post HLI | DNMAML non-ischemic adductor | 39.2852 | 9.9269 | 5 | **-** | μm | DNMAML non-ischemic to ischemic | Students’ t test, unpaired | Day7 | 2E | IHC expts. Used validated Abs. Representative images were used in manuscript |
|  |  | DNMAML ischemic adductor | 46.5264 | 6.7755 | 5 | **0.2195** | μm |  |  |  |  |  |
| 6. qRT-PCR of Notch targets relative to 18s post HLI in DNMAML | *Notch1, Jag1, Hes1* decreases following HLI in DNMAML | Compare ischemic to non-ischemic ECs |  |  | 3 |  | Fold change | WT ischemic / non-ischemic to  DNMAML  Ischemic / non-ischemic | Students’ t test, unpaired | Day5 sorted EC | 2F | Compared to Wt values in experimental questions #1 |
|  |  | *Notch1* | 1.9477 | 0.3408 | 3 | **0.0146** |  |  |  |  | 2F |  |
|  |  | *Dll4* | 0.8119 | 0.1857 | 3 | 0.1609 |  |  |  |  | 2F |  |
|  |  | *Jag1* | 0.6372 | 0.0448 | 3 | **0.0377** |  |  |  |  | 2F |  |
|  |  | *Hes1* | 0.6595 | 0.2407 | 3 | **0.0007** |  |  |  |  | 2F |  |
|  |  | *Hey1* | 0.5040 | 0.1116 | 3 | 0.7300 |  |  |  |  | 2F |  |
| 7. Measure of aEC recovery following HLI in DNMAML | aEC population fails to recover in DNMAML mice post HLI | Adductors of WT ischemic and non-ischemic tissue | 93.1761 | 7.9105 | 5 | **0.0038** | Percentage | WT ischemic / non-ischemic to  DNMAML  Ischemic / non-ischemic | Students’ t test, unpaired | Day5 sorted EC | 2G |  |
|  |  | DNMAML | 74.8097 | 3.7140 | 5 |  |  |  |  |  |  |  |
| 8. Assess perfusion recovery in NICD mice | NICD recovers perfusion  post HLI compared to DNMAML | Comparison of perfusion NICD vs DNMAML hind limbs | - | - | - | - | PU  Perfusion units | WT vs DNMAML | Multiple unpaired t-test. Holm-Sidak method | Days 1, 3, 5, 7 | 2C | observation |
|  |  | NICD | 12.6135 | 9.1874 | 8 | 0.999 | PU | + | + | Day1 | 2C |  |
|  |  | DNMAML | 9.5210 | 3.4286 | 8 |  | PU |  |  |  |  |  |
|  |  | NICD | 16.7564 | 6.8495 | 8 | 0.03595 | PU | + | + | Day3 | 2C |  |
|  |  | DNMAML | 11.1279 | 4.7492 | 8 |  | PU |  |  |  |  |  |
|  |  | NICD | 27.4062 | 6.2099 | 8 | **0.02740** | PU | + | + | Day5 | 2C |  |
|  |  | DNMAML | 19.6965 | 7.4624 | 8 |  | PU |  |  |  |  |  |
|  |  | NICD | 43.0921 | 11.1720 | 8 | **0.001563** | PU | + | + | Day7 | 2C |  |
|  |  | DNMAML | 24.4661 | 4.1812 | 8 |  | PU | + | + |  |  |  |
| 9. Measure NICD collateral artery diameter following HLI | NICD arteries  expanded compared to DNMAML  post HLI | NICD non-ischemic adductor | 36.3781 | 61.9584 | 5 | - | μm | DNMAML non-ischemic to ischemic | Students’ t test, unpaired | Day7 | 3E | IHC expts. Used validated Abs. Representative images were used in manuscript |
|  |  | NICD ischemic adductor | 8.5588 | 8.2098 | 5 | **0.0013** | μm |  |  |  |  |  |
| 10. qRT-PCR of Notch targets relative to 18s post HLI in NICD mice | *Dll4* and *Hey1* increases following HLI in NICD | Compare ischemic to non-ischemic ECs |  |  | 3 |  | Fold change | DNMAML ischemic / non-ischemic  NICD  Ischemic / non-ischemic | Students’ t test, unpaired | Day5 sorted EC | 3F | Compared to DNMAML values in experimental questions #6 |
|  |  | *Notch1* | 1.5012 | 0.2840 | 3 | 0.15623 |  |  |  |  | 2F |  |
|  |  | *Dll4* | 2.0026 | 0.4394 | 3 | **0.01241** |  |  |  |  | 2F |  |
|  |  | *Jag1* | 1.0246 | 0.2254 | 3 | 0.0432 |  |  |  |  | 2F |  |
|  |  | *Hes1* | 0.8393 | 0.2314 | 3 | 0.4037 |  |  |  |  | 2F |  |
|  |  | *Hey1* | 1.5766 | 0.1531 | 3 | **0.0006** |  |  |  |  | 2F |  |
| 11. Measure of aEC recovery following HLI in NICD | aEC population recover in NICD mice post HLI | Adductors of NICD ischemic and non-ischemic tissue | 92.7929 | 8.0215 | 5 | **0.0045** | Percentage | DNMAML ischemic / non-ischemic to  NICD  Ischemic / non-ischemic | Students’ t test, unpaired | Day5 sorted EC | 2G | Compared to DNMAML values in experimental questions #7 |
| 12. In-vivo validation of RNA seq targets | Sema3g and Hes1 are putative Notch targets | Adductors of WT, DNMAML, and NICD ischemic and non-ischemic tissue | - | - | 3 | **-** | Relative expression to *18s* | WT, DNMAML, and NICD | Observation | Day5 sorted EC | 4D | Characteristic higher expression in NICD and in WT than in DNMAML |
|  | WT | *Sema3g* | 0.1676 | 0.0115 | 3 | - | + |  | + | + | 4D |  |
|  | DNMAML | *Sema3g* | 0.0478 | 0.0199 | 3 | - | + |  | + | + | 4D |  |
|  | NICD | *Sema3g* | 0.2780 | 0.0439 | 3 | - | + |  | + | + | 4D |  |
|  | WT | *Hes1* | 0.1678 | 0.0192 | 3 | - | + |  | + | + | 4D |  |
|  | DNMAML | *Hes1* | 0.0812 | 0.0316 | 3 | - | + |  | + | + | 4D |  |
|  | NICD | *Hes1* | 0.0972 | 0.0267 | 3 | - | + |  | + | + | 4D |  |
|  | WT | *Cxcl5* | 0.0435 | 0.0100 | 3 | - | + |  | + | + | 4D |  |
|  | DNMAML | *Cxcl5* | 0.0101 | 0.0035 | 3 | - | + |  | + | + | 4D |  |
|  | NICD | *Cxcl5* | 0.0108 | 0.0040 | 3 | - | + |  | + | + | 4D |  |
|  | WT | *Aplnr* | 0.0100 | 0.0021 | 3 | - | + |  | + | + | 4D |  |
|  | DNMAML | *Aplnr* | 0.0065 | 0.0024 | 3 | - | + |  | + | + | 4D |  |
|  | NICD | *Aplnr* | 0.0013 | 0.0002 | 3 | - | + |  | + | + | 4D |  |
| 13. In-vitro validation of RNA seq targets via GSI treatment and washout in HPAECs | Sema3g, Hes1, Cxcl5, Aplnr all sensitive to GSI, but not characteristic Notch reponse | HPAECs treated with GSI | - | - | 3 | **-** | Relative expression to *18s* | Ctrl, GSI, and GSI washout | 1way ANOVA | 48hr GSI treatment | 4D | Characteristic suppression and rescue in HES1 and SEMA3G |
|  | Ctrl | *SEMA3G* | 0.0008 | 0.0001 | 3 | **<0.0001** | + |  | + | + | 4E |  |
|  | GSI | *SEMA3G* | 0.0478 | 0.0199 | 3 | **<0.0001** | + |  | + | + | 4E |  |
|  | WO | *SEMA3G* | 0.2780 | 0.0439 | 3 | **<0.0001** | + |  | + | + | 4E |  |
|  | Ctrl | *HES1* | 0.0037 | 0.0008 | 3 | **0.0012** | + |  | + | + | 4E |  |
|  | GSI | *HES1* | 0.0009 | 0.0001 | 3 | **0.0012** | + |  | + | + | 4E |  |
|  | WO | *HES1* | 0.0034 | 0.0005 | 3 | **0.0012** | + |  | + | + | 4E |  |
|  | Ctrl | *CXCL5* | 0.0005 | 0.0001 | 3 | **0.0263** | + |  | + | + | 4E |  |
|  | GSI | *CXCL5* | 0.0003 | 0.0001 | 3 | **0.0263** | + |  | + | + | 4E |  |
|  | WO | *CXCL5* | 0.0004 | 0.0001 | 3 | **0.0263** | + |  | + | + | 4E |  |
|  | Ctrl | *APLNR* | 0.0000 | 0.0000 | 3 | **0.0023** | + |  | + | + | 4E |  |
|  | GSI | *APLNR* | 0.0009 | 0.0001 | 3 | **0.0023** | + |  | + | + | 4E |  |
|  | WO | *APLNR* | 0.0006 | 0.0003 | 3 | **0.0023** | + |  | + | + | 4E |  |
| 14. Compare DNMAML HPAECs to ctrl GFP HPAECs’ phenotypes | DNMAML induce hypersprouting angiogenesis phenotype | DNMAMLEC spheroids | 29642.4566 | 15314.03 | 40 | **0.0147** | GFP signal in pixels | pCCL-GFP vs pCCL-DNMAML HPAECs | Students’ t test, unpaired | 3D collagen matrix | 5B | Data was shown as a percentage of total field of view |
|  |  | GFP EC Spheroids | 22498.764 | 11573.4018 | 40 | **-** | + | + | + | + | 5B |  |
| 15. Compare SEMA3G effects on Ctrl HPAECs | SEMA3G reduces spheroid sprouting | GFP EC Spheroids + SEMA3G | 14289.0608 | 5954.8108 | 40 | **0.0002** | GFP signal in pixels | pCCL-GFP vs  pCCL-GFP + SEMA3G | Students’ t test, unpaired | 3D collagen matrix | 5B | Data was shown as a percentage of total field of view |
| 16. Compare SEMA3G effects on DNMAML HPAECs | SEMA3G reduces spheroid sprouting in DNMAML | DNMAML EC Spheroids + SEMA3G | 21781.2339 | 14746.4319 | 40 | **0.0182** | GFP signal in pixels | pCCL-DNMAML vs  pCCL-DNMAML + SEMA3G | Students’ t test, unpaired | 3D collagen matrix | 5B | Data was shown as a percentage of total field of view |
| 17. Validate DNMAML HPAECs | DNMAL suppresses Notch Signaling in HPAECs | HPAECs overexpressing DNMAML |  |  | 3 | **-** | Relative expression to *18s* | HPAECs-GFP vs HPAECS-DNMAML | Students’ t test, unpaired | Confludent monolayer | 5C | HES1, NOTCH1, SEMA3G are affected by Notch signaling |
|  | HPAEC-GFP | *NOTCH1* | 0.0115 | 0.0016 | 3 | **0.0096** | + | + | + |  | 5C |  |
|  | HPAEC-DNMAML | *NOTCH1* | 0.0022 | 0.0001 | 3 |  |  |  |  |  | 5C |  |
|  | HPAEC-GFP | *HES1* | 0.0126 | 0.0014 | 3 | **0.0070** | + | + | + |  | 5C |  |
|  | HPAEC-DNMAML | *HES* | 0.0022 | 0.0001 | 3 |  |  |  |  |  | 5C |  |
|  | HPAEC-GFP | *SEMA3G* | 0.0025 | 0.0009 | 3 | **0.0398** | + | + | + |  | 5C |  |
|  | HPAEC-DNMAML | *S3MA3G* | 0.0002 | 0.0002 | 3 |  |  |  |  |  | 5C |  |
| 18. Assess expression of Semaphorin and VEGF receptors | Inhibition of Notch signaling increases *NRP1* and *NRP2* | HPAECs overexpressing DNMAML |  |  | 3 | **-** | Relative expression to *18s* | HPAECs-GFP vs HPAECS-DNMAML | Students’ t test, unpaired | Confludent monolayer | 5C | NRP1 and NRP2 are upregulated when Notch signaling is low |
|  | HPAEC-GFP | *NRP1* | 0.0045 | 0.0008 | 3 | **0.0072** | + | + | + |  | 5C |  |
|  | HPAEC-DNMAML | *NRP1* | 0.0185 | 0.0026 | 3 |  |  |  |  |  | 5C |  |
|  | HPAEC-GFP | *NRP2* | 0.0156 | 0.0012 | 3 | **0.0088** | + | + | + |  | 5C |  |
|  | HPAEC-DNMAML | *NRP2* | 0.0599 | 0.0077 | 3 |  |  |  |  |  | 5C |  |
|  | HPAEC-GFP | *PLXND1* | 0.0566 | 0.0036 | 3 | **0.0035** | + | + | + |  | 5C |  |
|  | HPAEC-DNMAML | *PLXND1* | 0.0073 | 0.0083 | 3 |  |  |  |  |  | 5C |  |
| 19. Measure vascular density of control and DNMAML retinas | DNMAML induces hypdersprouting in retinal vasculature | Mouse retinas |  |  | 12 | **<.0001** | Isolectin staining measured as pixels | Ctrl DNMAML vs DNMAML-CRE retinas | Students’ t test, unpaired | P6 mouse retinas | 6C | Isolectin stains microvasculature |
|  |  | Ctrl | 40.0631 | 8.4258 | + |  |  |  |  |  |  |  |
|  |  | DNMAML | 60.6266 | 7.6611 | + |  |  |  |  |  |  |  |
| 20. Measure filopodia in control and DNMAML retinas | DNMAML induces more sprouts in retinas | Mouse retinas |  |  | 12 | **<.0001** | Filopodia extension | Ctrl DNMAML vs DNMAML-CRE retinas | Students’ t test, unpaired | P6 mouse retinas | 6C |  |
|  |  | Ctrl | 22.8333 | 5.1493 | + |  |  |  |  |  |  |  |
|  |  | DNMAML | 36.5000 | 4.9082 | + |  |  |  |  |  |  |  |
| 21. Measure vascular density of DNMAML retinas with SEMA3G | SEMA3G reduces vascular spreading in retinal vasculature | Mouse retinas |  |  | 12 | **0.0048** | Isolectin staining measured as pixels | Ctrl DNMAML vs DNMAML-CRE retinas | Students’ t test, unpaired | P6 mouse retinas | 6C | Isolectin stains microvasculature |
|  |  | DNMAML | 61.7851 | 13.2515 | + |  |  |  |  |  |  |  |
|  |  | DNMAML+ SEMA3G | 50.4904 | 10.0384 | + |  |  |  |  |  |  |  |
| 22. Measure filopodia in DNMAML retinas with SEMA3G | SEMA3G inhibits sprout formation in DNMAML retinas | Mouse retinas |  |  | 12 | **0.0137** | Filopodia extension | Ctrl DNMAML vs DNMAML-CRE retinas | Students’ t test, unpaired | P6 mouse retinas | 6C |  |
|  |  | DNMAML | 27.2500 | 9.0667 | + |  |  |  |  |  |  |  |
|  |  | DNMAML+ SEMA3G | 17.5000 | 8.7542 | + |  |  |  |  |  |  |  |

*You may use multiple lines for the same question to indicate multiple comparisons

** Authors may wish to make the text bold where p is considered significant against a stated confidence limit.
